# Combination of µCT and light microscopy for generation-specific stereological analysis of pulmonary arterial branches: a proof-of-concept study

**DOI:** 10.1007/s00418-020-01946-x

**Published:** 2020-12-02

**Authors:** Roman Grothausmann, Jonas Labode, Pablo Hernandez-Cerdan, David Haberthür, Ruslan Hlushchuk, Oleg Lobachev, Christina Brandenberger, Andre George Gie, Thomas Salaets, Jaan Toelen, Willi L. Wagner, Christian Mühlfeld

**Affiliations:** 1 Institute of Functional and Applied Anatomy, Hannover Medical School, Carl-Neuberg-Str. 1, 30625 Hannover, Germany; 2 Biomedical Research in Endstage and Obstructive Lung Disease Hannover (BREATH), Member of the German Center for Lung Research (DZL), Hannover, Germany; 3 Faculty of Engineering and Health, HAWK University of Applied Sciences and Arts, Von-Ossietzky-Str. 99, 37085 Göttingen, Germany; 4 Phcerdan, Medical Image Analysis Research and Development, Torre-Pacheco, Spain; 5 Institute of Anatomy, University of Bern, Baltzerstrasse 2, 3012 Bern, Switzerland; 6 Department of Development and Regeneration, KU Leuven, Leuven, Belgium; 7 Department of Diagnostic and Interventional Radiology (DIR), University of Heidelberg, Heidelberg, Germany; 8 Translational Lung Research Center (TLRC), Member of the German Center for Lung Research (DZL), University of Heidelberg, Heidelberg, Germany

**Keywords:** Pulmonary vasculature, Branching generation analysis, Micro computed tomography, Stereology, 3D reconstruction, Light microscopy

## Abstract

Various lung diseases, including pulmonary hypertension, chronic obstructive pulmonary disease or bronchopulmonary dysplasia, are associated with structural and architectural alterations of the pulmonary vasculature. The light microscopic (LM) analysis of the blood vessels is limited by the fact that it is impossible to identify which generation of the arterial tree an arterial profile within a LM microscopic section belongs to. Therefore, we established a workflow that allows for the generation-specific quantitative (stereological) analysis of pulmonary blood vessels. A whole left rabbit lung was fixed by vascular perfusion, embedded in glycol methacrylate and imaged by micro-computed tomography (µCT). The lung was then exhaustively sectioned and 20 consecutive sections were collected every 100 µm to obtain a systematic uniform random sample of the whole lung. The digital processing involved segmentation of the arterial tree, generation analysis, registration of LM sections with the µCT data as well as registration of the segmentation and the LM images. The present study demonstrates that it is feasible to identify arterial profiles according to their generation based on a generation-specific color code. Stereological analysis for the first three arterial generations of the monopodial branching of the vasculature included volume fraction, total volume, lumen-to-wall ratio and wall thickness for each arterial generation. In conclusion, the correlative image analysis of µCT and LM-based datasets is an innovative method to assess the pulmonary vasculature quantitatively.

## Introduction

The mammalian pulmonary vasculature consists of an arterial inflow, a vast capillary bed and one or more veins draining the blood from the lungs. To supply the large amount of capillaries, the pulmonary arteries form a tree-like structure with numerous branches of decreasing diameter. In principle, the arterial branches show the same geometric branching pattern as the airways which they accompany, whereas, in the peripheral parts of the lung, the veins run separately from arteries and airways. Small arteries branching off in an irregular and more or less perpendicular fashion are known as supernumerous arteries but are rather an exception from the general branching pattern. In larger animals including humans, the general branching pattern of airways and arteries is mainly dichotomous; whereas in smaller animals, like rodents or rabbits, a monopodial branching pattern prevails (Ochs and Weibel 2008; Singhal et al. 1973; Townsley 2012; Horsfield 1978, 1984).

As the pulmonary vasculature and, particularly the arterial system, contributes to several human lung diseases, including pulmonary arterial hypertension (Tuder 2017), chronic obstructive pulmonary disease (Kasahara et al. 2001), diffuse parenchymal lung disease (Ackermann et al. 2020a), COVID-19 pneumonia (Ackermann et al. 2020b) or bronchopulmonary dysplasia (Thébaud and Abman 2007; Jiménez et al. 2016), qualitative or quantitative microscopic investigations of the pulmonary arteries are vital for our pathophysiologic understanding. One of the main problems of these analyses is the question how to define a specific vascular compartment (Mühlfeld et al. 2018). The arterial tree consists of a varying number of branching generations which may differ with respect to their wall composition or behavior in health and disease. Therefore, it would be desirable to analyze the arterial generations separately or to choose a specific generation of interest for the analysis. However, a large amount of arterial profiles can usually be seen in a single two-dimensional section through the lung without the investigator being able to assign the profile to a certain location within the vascular tree. Approaches to overcome this problem are, for example, microdissection (Davies and Reid 1991) or corrosion casts (de Mello et al. 1997) of the vascular tree or a compartmentalization of the arteries based on their diameter or wall characteristics (Mühlfeld and Ochs 2013). Microdissection has the disadvantage that the surrounding tissue is irrevocably lost and a certain size limit prohibits the analyses of smaller branches. Corrosion casts are suitable to investigate the branching pattern and geometry of the vasculature but the loss of surrounding tissue makes it impossible to study cellular characteristics. Finally, a wall composition- or diameter-based approach carries the potential of being severely confounded by changes in the wall characteristics due to disease development.

Hence, in the present study, we established a new approach of combining three-dimensional knowledge of the vascular tree with subsequent generation-specific microscopic analysis of the pulmonary arteries. The new technique relies on a workflow involving µCT of an embedded sample, segmentation of the arterial tree, exhaustive cutting of the sample for LM, registration of digitalized microscopic images with reconstructed µCT slices and finally, assignment of the branching generation to specific arterial profiles. As a by-product, the same information can be used for analysis of airway generations.

## Materials and methods

### Rabbit lung

The rabbit lung used in this study was part of a larger set of experiments on bronchopulmonary dysplasia. The experiments were approved by the Ethics committee for Animal Experimentation of KU Leuven, Belgium, (P081/2017) and conform to national and international laws for animal welfare. In brief, the experiments included preterm delivery by cesarean section at 28 days of pregnancy (term = 31 days), seven days in an incubator at 32 °C, 50% humidity and exposure to hyperoxia (≥ 95% oxygen). Deep anesthesia was induced with 35 mg/kg of ketamine and 6 mg/kg of xylazin. The trachea was exposed to insert a cannula attached to a ventilator delivering first a recruitment manoeuver and then a steady ventilation with a tidal volume of 8 ml/kg at 120 breaths/min and with a positive end-expiratory pressure (PEEP) of 10 cm H_2_O. Whilst on the ventilator a laparotomy was performed, the diaphragm was opened and the heart was visualized by bilateral thoracotomy. After repetition of the recruitment manoeuver with an open chest, the trachea was tied of at a PEEP of 10 cm H_2_O. Immediately, the right ventricle was catheterized to perform intravascular fixation of the lungs. During vascular perfusion, the deeply anesthetized rabbit is euthanized by exsanguination. Vascular perfusion included a saline pre-flush before fixation at a pressure of 30 cm H_2_O using a 4% paraformaldehyde fixation solution for 1 h. The heart–lung block was excised, stored in the same fixative at 4 °C till further processing.

### Lung processing

The left lung was separated from the heart–lung block and embedded in toto in glycol methacrylate (Technovit 7100, Heraeus Kulzer, Wehrheim, Germany). Briefly, the lung was postfixed with 1% osmium tetroxide and 1% uranyl acetate subsequently, dehydrated in an ascending acetone series and finally embedded in glycol methacrylate. Before embedding, the lung was rotated around a vertical axis through apex and base of the lung to allow the generation of vertical sections (Baddeley et al. 1986).

### Micro-computed tomography

The sample was imaged on a SkyScan 1272 high-resolution microtomograph (Control software version 1.1.19, Bruker microCT, Kontich, Belgium). The machine is equipped with a Hamamatsu L11871_20 X-ray source and a XIMEA xiRAY16 camera. The X-ray source was set to a tube voltage of 80.0 kV and a tube current of 125.0 µA, the x-ray spectrum was filtered by 1 mm Al prior to incidence onto the sample. We recorded a set of 2 stacked scans overlapping the sample height, each stack was recorded with 488 projections of 3104 × 1091 pixels (2 projections stitched laterally) at every 0.4° over a 180° sample rotation. Every single projection was exposed for 2247 ms; 5 projections were averaged to one to greatly reduce image noise. This resulted in a total scan time of approximately 8 h. The projection images were then subsequently reconstructed into a 3D stack of images with NRecon (Version 1.7.4.2, Bruker microCT, Kontich Belgium) using a ring artifact correction of 7. The whole process resulted in datasets with an isometric voxel size of 7.0 µm.

### Segmentation and branching generation analysis

**Fig. 1:**
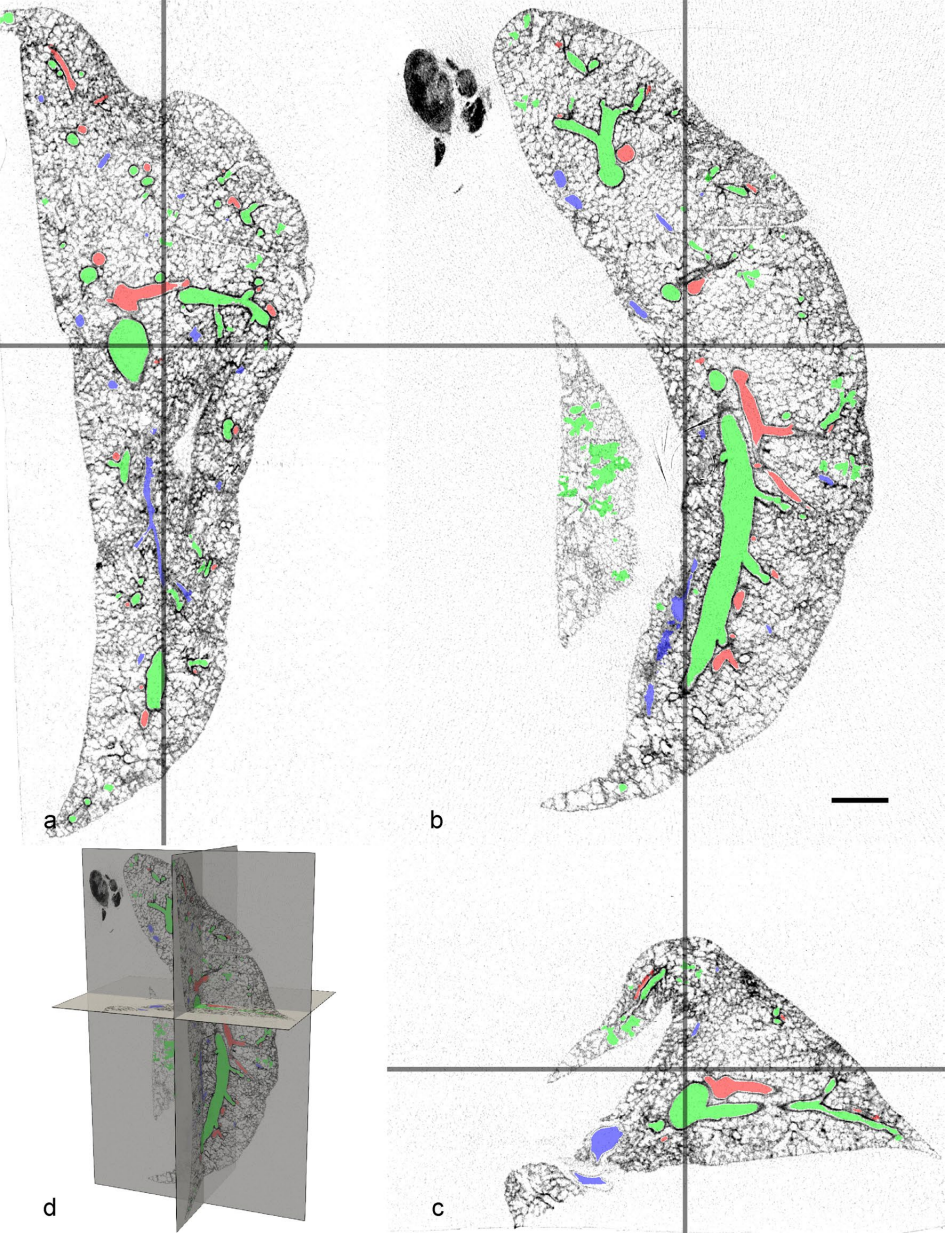
**a**–**c** slices through the (inverted) µCT dataset in all three directions overlayed with the segmentation of the arteries (red), airways (green) and veins (blue). **d** 3D arrangement of the slices. Scale bar = 1 mm

**Fig. 2:**
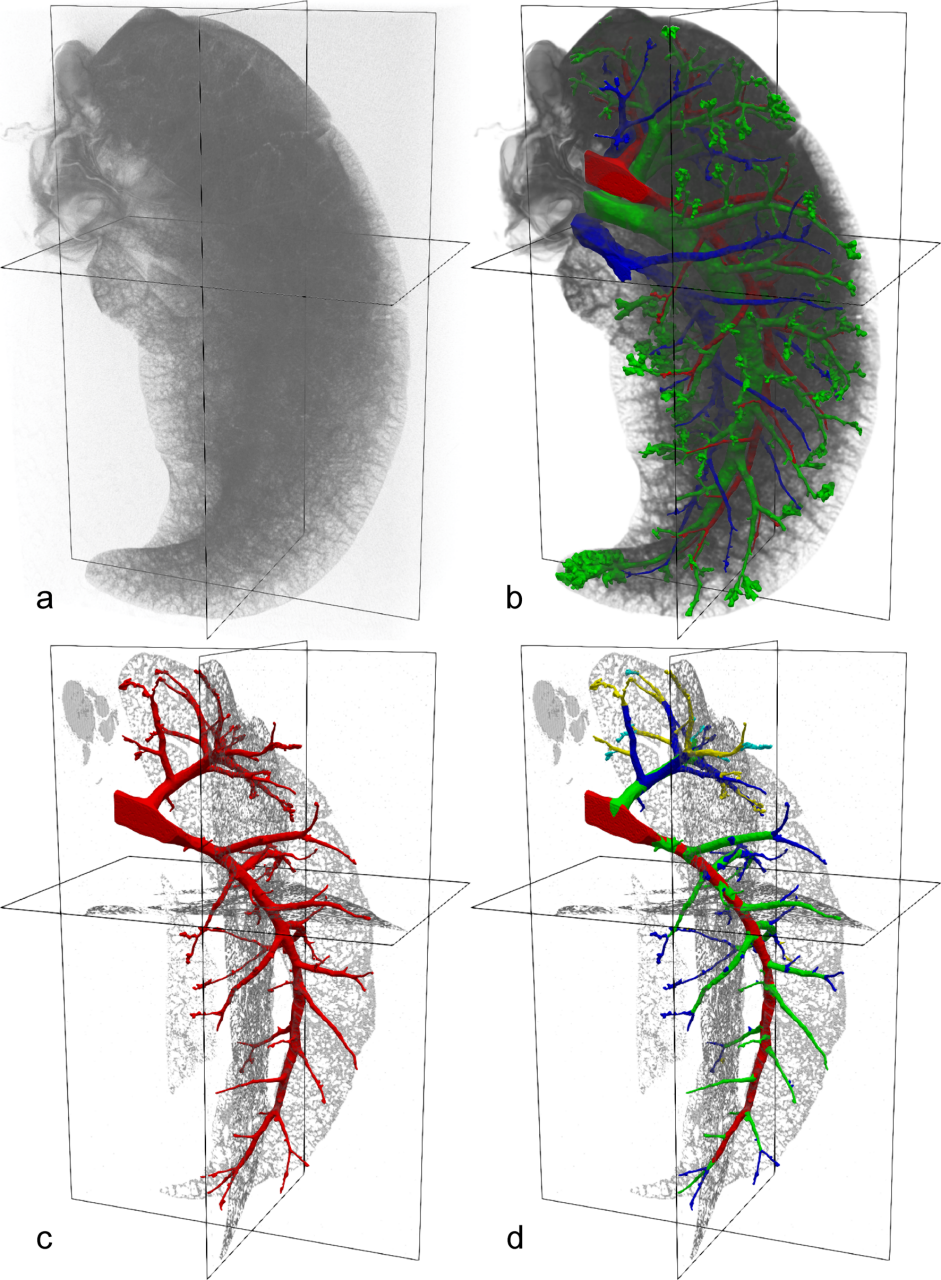
**a** Volume rendering of the (inverted) µCT dataset, **b** volume rendering of the denoised µCT dataset and its segmentation, **c** only the artery segmentation and slices from the µCT dataset (as in Fig. 1d), **d** 3D visualization of the extracted arterial generations

The µCT dataset (see Fig. 1) was denoised with a 3D anisotropic gradient diffusion filter (compare Fig. 2a, b). The resulting dataset was used to manually create lids for the artery, vein and airway in ITKSnap (Yushkevich et al. 2006) with the adaptive brush. These lids were used to close the opened vessels and airways, to allow generating a pre-segmentation based on the gradient magnitude image followed by a 3D morphological watershed transform. This pre-segmentation was used as a base in an extended version of ITKSnap to assign segments to either arteries (red), veins (blue) or airspace (green) with the Click’n’Join mode (for more details see https://github.com/pyushkevich/itksnap/pull/1, see Figs. 1, 2b and 4a). Since arteries are the main focus of this study, veins and conducting airspaces were only segmented to avoid confusion with other tubular structures and their cross sections (leaving the airspace segmentation incomplete, blood vessels were segmented as far as the resolution allowed).

The segmentation of the arterial tree was extracted for the generation analysis, Figs. 2c, 4b. The semi-manual generation analysis is also realized with the Click’n’Join mode of the extended version of ITKSnap. The needed pre-segmentation was created from a voxel skeleton (centerlines, created by SGEXT[https://github.com/phcerdan/SGEXT]), processes such that each branch has its own label and then spread by a 3D morphological watershed to extent over the artery segmentation. Each branch was then assigned to its monopodial branching generation (from 1st to 5th with red, green, blue, yellow, cyan, see Figs. 2d, 4cd).

Automated digital image processing was done with command-line interfaces [ITK-CLIs (https://github.com/romangrothausmann/ITK-CLIs), VTK-CLIs(https://github.com/romangrothausmann/VTK-CLIs)] and Python scripts (see https://gitlab.com/romangrothausmann/bpd-vesgen/-/blob/master/.gitmodules for details) based on ITK [http://www.itk.org] (Ibanez et al. 2020), VTK [http://www.vtk.org] (Schroeder et al. 2006), SimpleElastix [https://simpleelastix.github.io/] (Marstal et al. 2016), SGEXT[https://github.com/phcerdan/SGEXT] and ParaView [http://www.paraview.org] (Ayachit 2016). The processing dependencies are defined in Makefiles (gnu-make [https://www.gnu.org/software/make/] and gnu-parallel [http://www.gnu.org/s/parallel] (Tange 2011)), https://gitlab.com/romangrothausmann/bpd-vesgen/. Their evolution was tracked with git (https://git-scm.com/) and git-annex (https://git-annex.branchable.com/) was used for raw, intermediate and final result files.

### Lung sectioning

The lung was exhaustively sectioned parallel to the vertical axis (see above). Starting with a random number between 1 and 100, 20 consecutive sections of 2 µm thickness were collected and mounted on glass slides (each regarded as a substack). The next 50 section (100 µm) were discarded and then again 20 sections were mounted on glass slides. This procedure was repeated until the whole lung was sectioned. The sections were then stained with toluidine blue and digitalized using a Zeiss Axioscan (Zeiss, Göttingen, Germany) slide scanner.

### 3D reconstruction of light microscopic sections and 3D registration with µCT

**Fig. 3:**
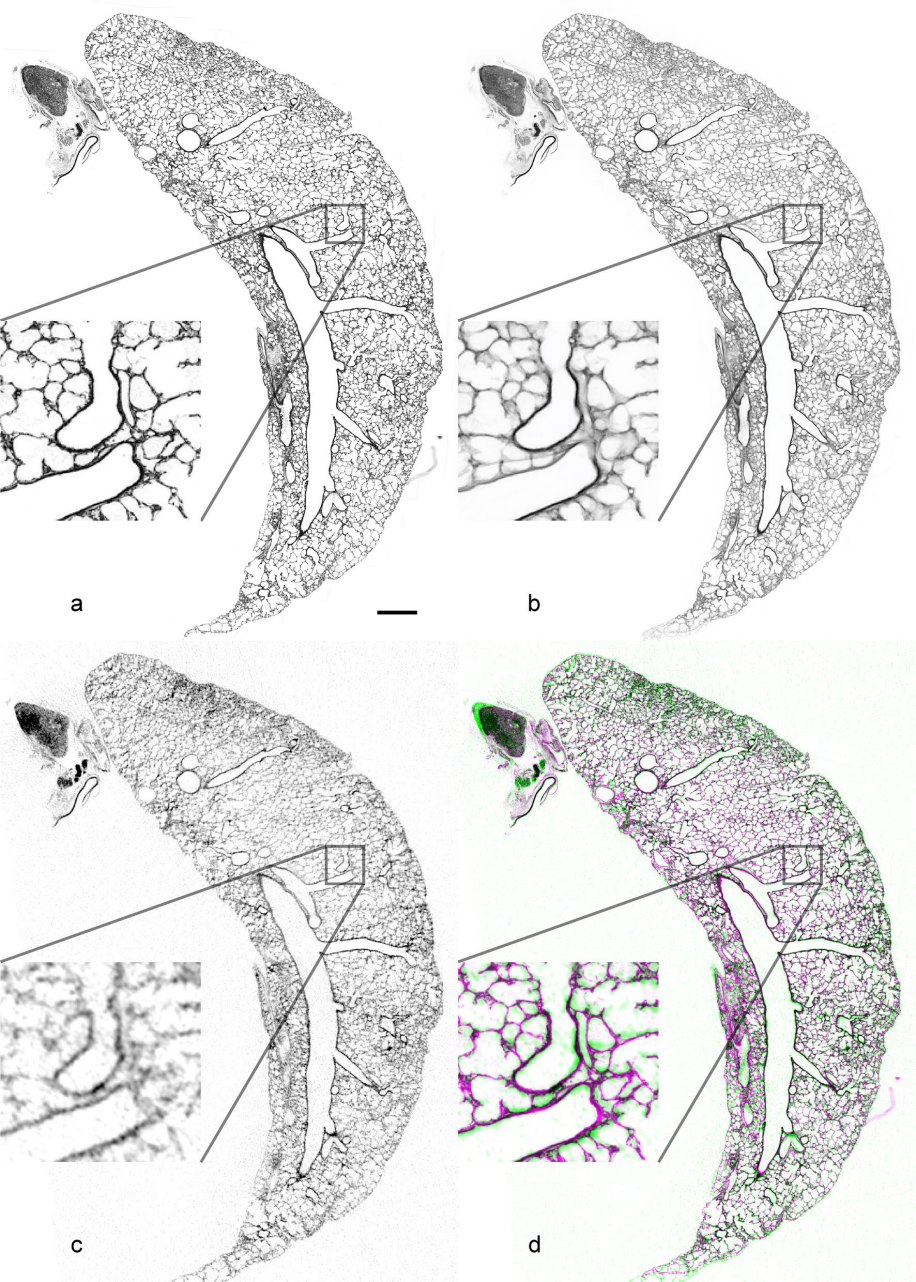
**a** slice of an LM substack (converted to gray values) similar to that of Fig. 1b , **b** z-projection of the whole corresponding LM substack (in-plane septa and nearly fully contained vessels are now visible), **c** corresponding slice of the registered µCT dataset, **d** RGB overlay of the LM slice (as shown in **a**), green channel and the µCT slice (as shown in **c**), magenta channel, matching regions appear gray, whereas discrepancies appear green/magenta. Scale bar = 1 mm. Insets show a × 5 magnification

The images of the digitalized serial sections in CZI-format were processed with CZIto3D (http://github.com/romangrothausmann/CZIto3D.git) and aligned to each other with recRegStack.py (http://github.com/romangrothausmann/elastix_scripts.git), see Grothausmann et al. (2020) for details. The 2D registrations of consecutive image pairs uses an affine transform to optimize translation, rotation, scale and shear parameters before employing a bspline transform to correct local deformations introduced by the physical cutting and histological staining. A resulting slice and the z-projection of the corresponding aligned substack are shown in Fig. 3a, b, respectively.

**Fig. 4:**
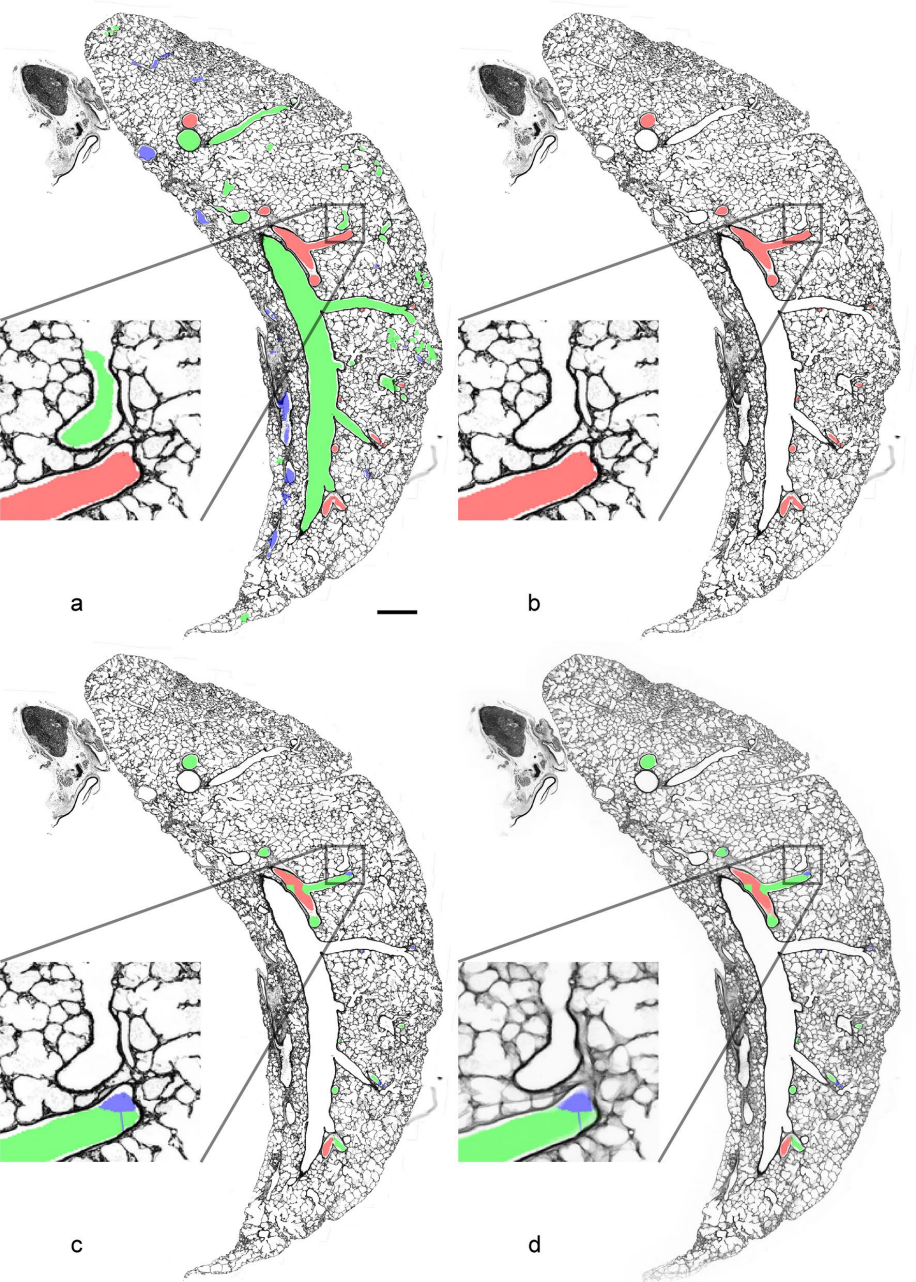
**a** LM slice (as in Fig. 3) overlayed with the transformed segmentation of the µCT dataset (as shown in Figs. 1, 2b), **b** same slice showing only the arterial segmentation, **c** overlay of the generation analysis of the arterial segmentation, **d** z-projection of the whole corresponding LM substack, changes of generations at junctions contained within the substack are now better visible. Scale bar = 1 mm. Insets show a 5 × magnification

The µCT dataset was then registered in 3D (affine and bspline) to each LM substack [footnote: Registering the LM substacks to the µCT is possible as well but leads to LM slices where regions originate from different physical section, which is non-ideal for the stereological evaluation], such that each LM slice has a corresponding µCT slice, see Fig. 3c, d. These transformations then allow to transform the segmentations and the result from the generation analysis (both done on the µCT dataset) to the LM substacks, of which slices were then used for the stereological evaluation, see Fig. 4.

### Stereology

The registered slices with colour-coded arterial branches were subjected to systematic uniform random sampling to determine the volume fraction of the first three arterial generations (red, green and blue) related to the volume of the lung. In addition, the volume fraction of arterial wall and lumen as well as the luminal surface area of the endothelial wall layer was estimated. For this purpose, a coarse grid with two points and a fine grid with 16 points were projected onto the images. Furthermore, a grid consisting of 36 line segments (length of test line per end point, *L*(point) = 52 µm) for vertical sections was used, i.e., a cycloid line system was used. The coarse grid was used to count points hitting the reference volume, here consisting of parenchymal and non-parenchymal parts of the lung. The fine grid was used to count points hitting the arterial branches (wall and lumen). Using the line segments, end points of the lines were counted if they hit wall structures or the lumen of the arterial branches. Intersections of the lines with the luminal surface of the arteries were also counted. The resulting counts were used to calculate the parameters of interest. Volume fractions of the first, second, and third generation arteries were calculated by *V*_V_(artery/lung) = *P*(artery)/(*P*(ref) × 8). The factor of 8 in the denominator originates from the fact that each point of the coarse grid represents eight points of the fine grid. The volume fraction of arterial wall and lumen was calculated by *V*_V_(wall/artery) = *P*(wall)/*P*(artery) and *V*_V_(lumen/artery) = *P*(lumen)/*P*(artery), respectively, where *P*(artery) is the count from the endpoints of the lines and differs from *P*(artery) in the formula above. The point and intersection counts were used to calculate the thickness of the wall by *T*(wall) = (*P*(wall) × *L*(point))/(2 × I).

## Results

Figure 1 shows exemplary 2D images of the µCT scan in three different orthogonal planes, images were inverted to match LM appearance. The segmentation of airways, arteries and veins is color coded, i.e., airways are labeled green, arteries red and veins blue. Due to the larger diameter of the airways, the segmentation reaches deeper into the periphery of the lung than that of arteries and veins. In Fig. 2, a volume rendering of the CT scan is shown with and without the segmentation. In Fig. 2b, the segmentation for airways, arteries, and veins are shown together. Airways and arteries are running closely together whereas only the large longitudinal vein is adjacent to the corresponding longitudinal main airway and artery. The next generation, however, shows a regular distribution pattern with the veins running in the middle between two airway/artery branches and alternate between the two sides of the main airway/artery. Figure 2c shows the isolated segmentation of the arterial tree before analysis of its generations. In Fig. 2d, the arteries have been assigned to generations. The main longitudinal artery is considered as generation 1 (red) and gives rise to appr. 20 arteries of generation 2 (green) which further divide into generation 3 (blue) arteries. Only in some cases, further branches could be assigned to generation 4 (yellow) or generation 5 (cyan) arteries, with their reduced number limiting further analysis of these generations.

Figure 3 presents the results of the registration of subsequent LM image pairs and of the µCT dataset to the corresponding 3D LM substack. Figure 3a shows one exemplary image of a longitudinal section through the whole lung with a thickness of 2 µm; whereas in Fig. 3b, 20 registered consecutive 2 µm thick sections are projected to form an image of a 40 µm thick substack. In Fig. 3c, a slice of the registered µCT dataset is shown which corresponds to the LM image presented in Fig. 3a. Corresponding LM image and µCT slice were overlaid to visualize the registration quality (Fig. 3d).

**Fig. 5:**
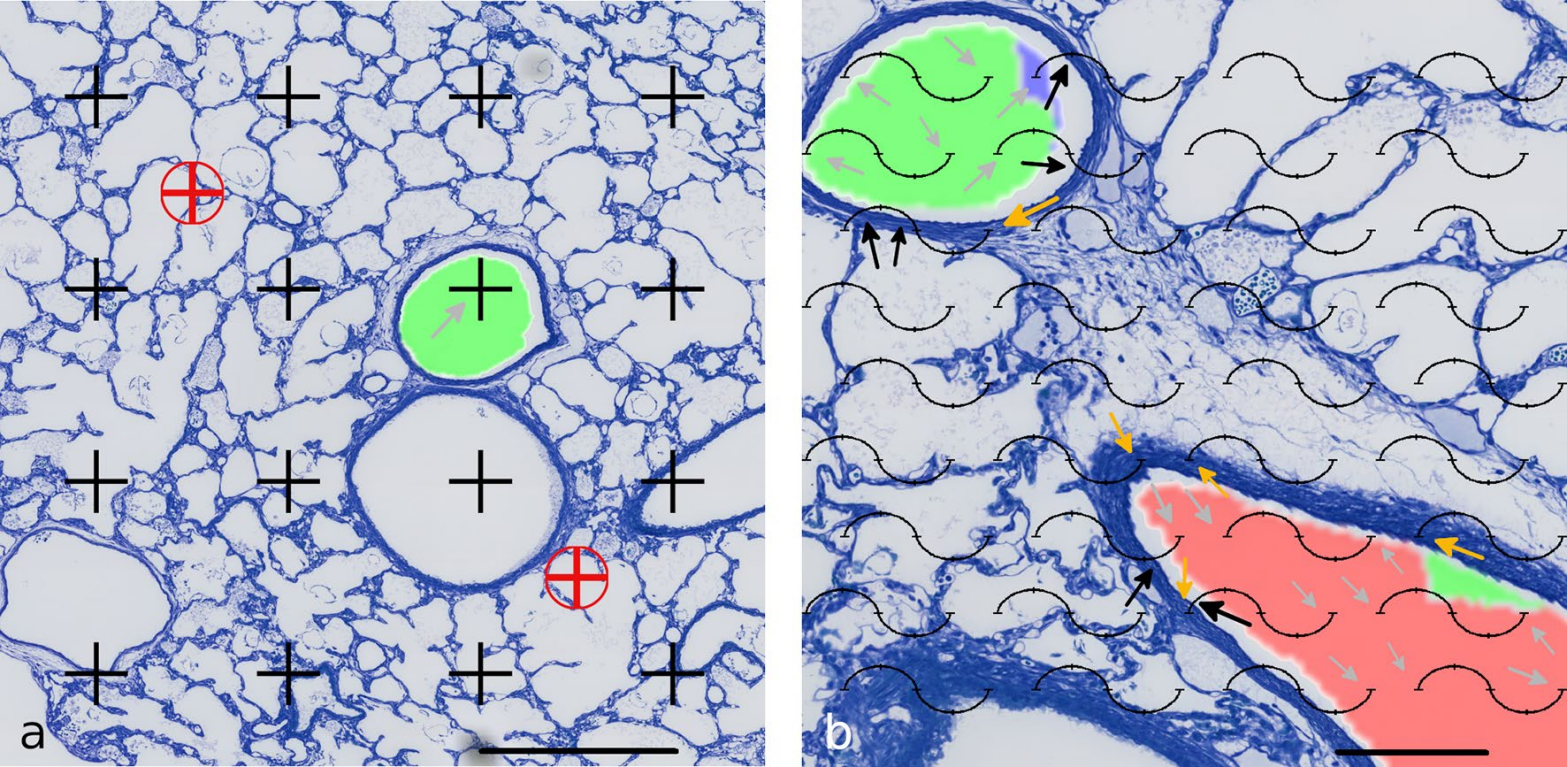
Stereological counting procedure. **a** A fine and coarse point grid was projected on low magnification LM images to estimate the volume fraction of arteries of specified generations related to the whole lung as reference volume. Here, 1 (arrow) of 16 black points of the fine grid hits the profile of a generation 2 artery (green), whereas 2 of 2 red points of the coarse grid hit the reference volume. One red point represents 8 black points. Scale bar = 850 µm. **b** A cycloid test system was projected on LM images at higher magnification to estimate the lumen-to-wall ratio and the arithmetic mean thickness of the wall. Counting events were intersections of the cycloids with the luminal boundary of the artery (black arrows), points hitting arterial wall (yellow arrows), points hitting arterial lumen (gray arrows). Note that the end points of the cycloids are used as a point grid and counting events are noted separately for different arterial generations. Here, for the green generation 2 artery, 4 intersections, 1 wall point and 6 lumen points would be counted, for the red generation 1 artery, 2 intersections, 4 wall points and 9 lumen points would be counted. Scale bar = 200 µm

The segmentation of airways, arteries and veins and the generation analysis from the µCT data were transformed to match with the LM substack (Fig. 4), thus enabling stereological analysis of arterial profiles according to their affiliation with a certain generation. The results of the stereological analysis of the first three generations are listed in Table 1. They illustrate some examples of useful parameters that can be estimated with the new method generation-wise. For a better understanding of the applied stereological tools, Fig. 5 shows a point grid and a cycloid test system that can be used for estimating the parameters shown in the table.

**Table 1:** Stereological data

	Generation 1 (red)	Generation 2 (green)	Generation 3 (blue)
*V*_V_ (artery/lung) [%]	0.320	0.446	0.287
*V* (artery, lung) [mm^3^]	0.682	0.951	0.612
lumen-to-wall ratio	4.21	3.91	4.855
*T* (wall) [µm]	23.8	16.3	12.1

## Discussion

**Fig. 6:**
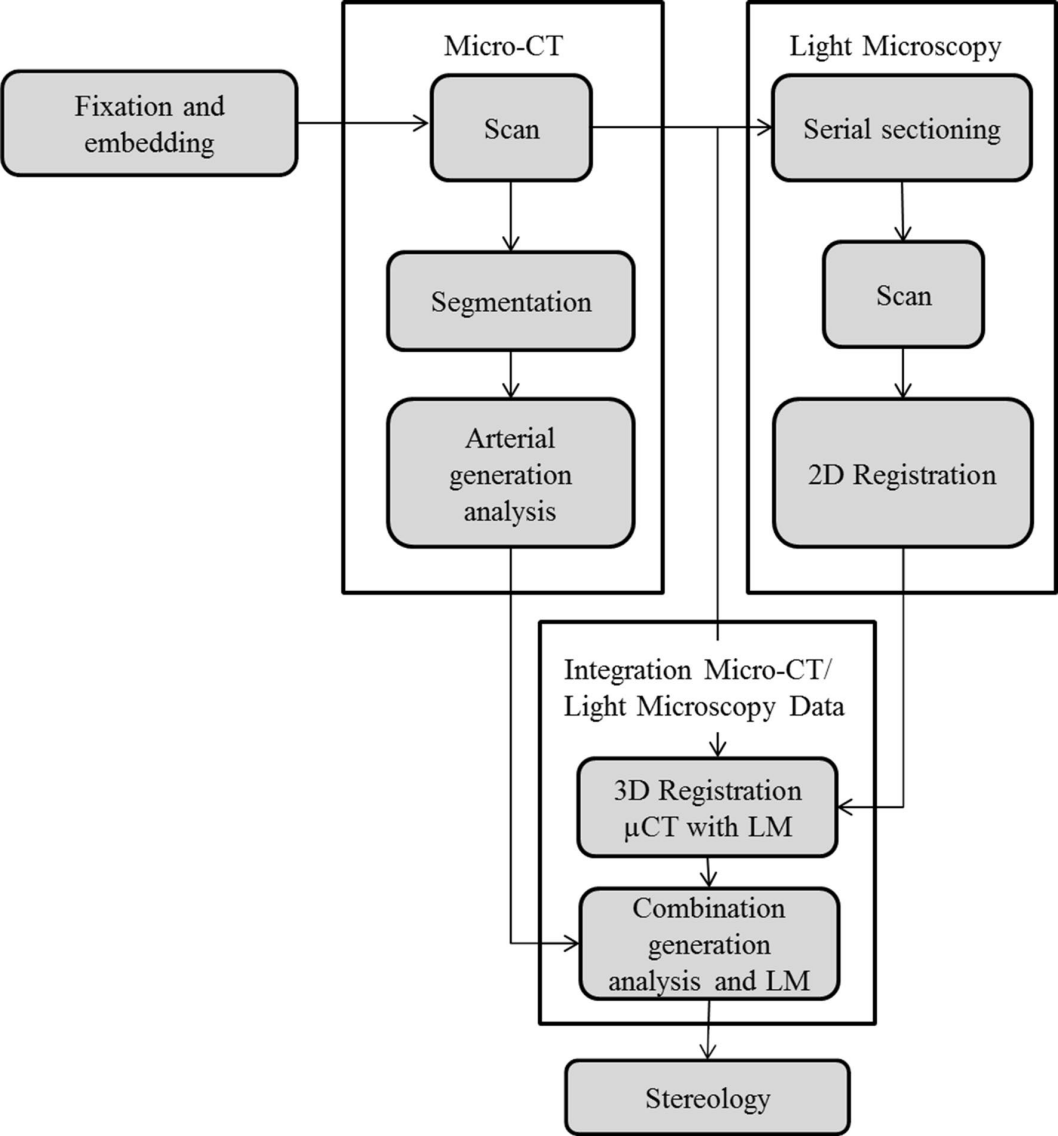
The proposed workflow as used in this study. Note: While the results of the µCT do not enter the histological serial sectioning process, there is a temporal dependency between these two steps. As the histological process is destructive, it can only start after the tasks that rely on an intact organ have been completed. All dependencies concerning the digital image processing are implied by the makefiles (see Material and Methods for details)

The aim of the present study was to establish a cross-scale bimodality imaging pipeline and a workflow that enables the analysis of pulmonary arterial branches quantitatively according to their generation and location within the arterial tree. The proposed protocol (Fig. 6) consists of the consecutive application and correlation of µCT and LM of the same sample, in this case a complete left lung of a postnatal rabbit.

The first step is the fixation of the lung which in principle can be performed by instillation (fixation via the airways) or by perfusion (fixation via the pulmonary arteries) (Weibel 1984). Although both types of fixation offer good tissue preservation, perfusion fixation was the method of choice in this study because it results in empty (i.e., free of erythrocytes) and open blood vessels (Buchacker et al. 2019). Both of these conditions are necessary for the digital reconstruction of the arterial tree; however, they are difficult to achieve within the whole lung. During perfusion fixation, a certain pressure needs to be maintained in the airways, which needs to be high enough to prevent the collapse of alveoli and low enough to allow the perfusate to flow through the blood vessels. Thus, the ratio between airway and perfusion pressure determines the quality and, therefore, the suitability of the sample for the subsequent analysis (Gehr et al. 1993; Gil 1990). In this study, a PEEP of 10 cm H_2_O on the deflation limb of the pressure–volume curve and a vascular perfusion pressure of 30 cm H_2_O yielded good results but this may vary in different species, in different developmental stages or in lung disease models that change the physical features of the lung.

The lung was then embedded in glycol methacrylate according to a slightly modified standard protocol (Schneider and Ochs 2014) with increased incubation times to compensate for the increased diffusion distances due to large sample size. The lung was embedded in an orientation that allows the generation of vertical sections with the vertical axis running from the apex of the lung to its base (Baddeley et al. 1986). For stereology of surface area and length either the sections or the combination of sections and test system have to be isotropic (Ochs and Mühlfeld 2013). Therefore, the lung was rotated along the vertical axis before embedding, which provides randomization of orientation in two dimensions.

After embedding, the lung was imaged using a µCT (Fig. 1). The settings were adjusted to provide the best possible contrast between empty airways, blood vessels and the surrounding tissue. As segmentation of the µCT dataset is based on the distinction between gray values, the contrast differences between tissue on the one hand and the glycol methacrylate-filled airspaces/blood vessels on the other hand greatly determines the success of the segmentation procedure. In this case, the segmentation of the arteries yielded complete results to the third branching generation. Further generations could be segmented but not necessarily completely. This procedure, however, is highly time-consuming and depends on the ability of the observer to identify profiles belonging to the same structure of interest. As can be seen from Fig. 2, the segmentation of the arteries was near to complete for the first three generations (red, green and blue) but only a few smaller arteries (generation 4 and 5, labeled with yellow and cyan) could be segmented which limits their quantitative analysis. These difficulties were similar for veins but less pronounced for airways. Possibly, the use of suitable contrast agents after perfusion fixation may help to enable the segmentation of smaller arteries as well (Hlushchuk et al. 2018; Faight et al. 2017; Jiménez et al. 2016). Furthermore, any increase in spatial resolution and/or signal to noise ratio (SNR) may improve segmentation results. Alternative modalities for non-destructive tomographic image acquisition include, e.g., synchrotron radiation-based imaging (Saccomano et al. 2018); however, the resulting increase in image data size will go along with higher demands to computational power and an increase in processing time. Nevertheless, due to the mainly monopodial branching pattern of rabbit lung airways and arteries, the resulting segmentation reaches deeply into the lung. In mainly dichotomously branched lungs, such as in humans, this would be equivalent to a much higher generation number.

In general, two ways of classifying the branches are used: “generations” are counted from the hilus (Ochs and Weibel 2008), whereas “orders” are counted starting in the periphery (Horsfield 1984). Both of these classifications are used for the human lung and have advantages and disadvantages. In a dichotomous lung, a new generation starts at every point where a mother branch divides into two daughter branches. For a monopodial branching pattern as present in the rabbit lung, the definition of generations has to be different. Various approaches to this have been proposed for the tracheobronchial tree (Lee et al. 2011; Madl et al. 2010; Wang and Kraman 2004). The branching pattern, seen in this study, corresponds to the lateral branching pattern described by Wang and Kraman for the canine airways (2004). In contrast to that study, here a modified classification was used with the longitudinal artery running through the whole left lung as generation 1, arteries laterally branching off from the generation 1 artery as generation 2, every artery branching off of a generation 2 artery as generation 3 and so forth. It has to be noted, however, that our classification does not take into account whether a certain artery is a proximal or distal branch of one generation. Future studies will need to provide a comparison of different branching classifications to allow grouping biologically similar arteries into one generation, possibly taking into account the geometrical distance from the hilus.

Stereology has been performed on µCT data previously (Vasilescu et al. 2012, 2013, Haberthür et al. 2013), the only factor limiting the applicability is the resolution required for certain parameters of interest. It needs to be emphasized that neither the µCT nor the LM resolution used in this study is at the maximal edge of what is possible. However, the µCT settings are close to the maximal resolution whereas the LM objective lens magnification corresponds to 10 × only and therefore has a lot of space for increasing the resolution. As shown in the insets of Fig. 3c, d, at the chosen resolution, the LM was superior to the µCT and the difference becomes more clearly visible in Fig. 5 where a 20 × LM magnification was used. Therefore, the stereological analysis of the blood vessels in this study was performed on the scanned LM sections which provides more cellular information than the µCT scan. For this purpose, the µCT dataset was registered with each substack (each consisting of 20 consecutive sections). Physical sections contain a number of deformation artifacts that change the sample dimensions such as compression, stretching or even distortion and make the registration challenging. Besides rotation and translation, also scale, shear and local deformation had to be adjusted. When applying these kinds of transformation, one has to be aware that these can change the extents of the structures contained in the section; hence, it changes the quantitative characteristics and introduces a potential bias which is inherent in histological sections. It would be possible to register the LM substacks to the µCT data to correct their deformations to the state before destructive processing, but due to the local deformation correction, larger substack depths would be needed to still be able to obtain LM slices from the corrected substacks containing the full sample cross section within a flat plane. These LM slices would in general contain data from multiple physical sections. Sampling LM slices from curved planes such that only data from the corresponding physical section is contained would become very difficult due to the correction of local deformations.

An alternative to the proposed combination of the generation analysis on the µCT matched to the LM substacks would be the segmentation solely from a complete set of digital images from serial sections through the whole lung. This approach would overcome the limitations by the reduced µCT resolution and would allow to segment vessels of smaller diameter up to the alveolar capillary network (Grothausmann et al. 2017). Initially, this approach was tested but failed because of several problems associated with sectioning artifacts. Loss of sections, section deformation or damage made it impossible to register the large amount of serial sections (appr. 2000 for a lung of comparable size) efficiently and coherently. Thus, the proposed protocol appeared to be the best compromise between time-efficiency and quality of results.

Design-based stereology is the gold standard of lung morphometry and has been reviewed in detail in the past years (Hsia et al. 2010,; Mühlfeld and Ochs 2013; Ochs and Mühlfeld 2013; Brandenberger et al. 2015). The stereological procedure used in this study provides an example of simple and easy-to-apply analyses of vascular features for different arterial generations, namely total volume, wall-to-lumen ratio and wall thickness. Without going into detail, some aspects relevant for the interpretation of the data need to be mentioned. First, the sections used for stereological analysis need to be randomly taken from the whole lung to guarantee equal representation of the arteries from every part of the lung (Mayhew 2008). In this case, a systematic uniform random approach (Gundersen and Jensen 1987) was used for the generation of the sections by starting with a random number for the first set of serial sections and then every further set of sections was taken at a given, predefined interval. Systematic uniform random sampling was also used for the selection of test images from the sections. Second, as mentioned above, the total parameter of the volume of an arterial generation depends on the quality and completeness of the segmentation. Incomplete segmentation would lead to a severe bias of the data when referred to total lung volume. Nevertheless, the wall-to-lumen ratio and the wall thickness are independent of the reference volume and can also be estimated from an incompletely segmented generation of arteries—if the reasons for incompleteness are not based on a systematic bias favoring the segmentation of a particular artery type. Third, vertical sections were used in this study because it decreases the work load of sectioning and facilitates the registration. However, one should keep in mind that vertical sections require a cycloid test system for the estimation of surface area (Baddeley et al. 1986). In addition to the estimated parameters, both from the µCT (e.g., vessel number, diameter or length) as well as from the LM (e.g., number of vascular wall smooth muscle cells or volumes of wall components) are possible. The latter, however, requires scanning the images at higher magnification, such as 40x, and potentially a more sophisticated staining.

At the current stage, the time required for the whole procedure of the proposed method precludes it from being used in a large series of experiments. The acquisition of the µCT data is only in the range of hours. With a duration of a few weeks, the contribution of the segmentation and generation analysis as well as the alignment, transformation and registration of the LM sections to the µCT data is the most time-consuming step. The stereological part including the cutting and scanning of the sections takes approximately a week; whereas, the actual counting can be done in a few hours. However, since the computational part of the method is most time-consuming, it can be expected that further progress in hard- and software will accelerate and automate the computing steps in the near future. In conclusion, we have established a new workflow for the generation-specific stereological analysis of pulmonary arteries combining non-destructive µCT imaging, segmentation, and LM stereology. Obviously, the procedure shown here for arteries can be used for airways and veins as well and will hopefully find its way into future pulmonary research.

## Availability of data and material

The processing dependencies are defined in Makefiles. The raw data can be provided upon request.

## Data Availability

Software is open source and freely available.
